# Effect of single-patient room design on the incidence of nosocomial infection in the intensive care unit: a systematic review and meta-analysis

**DOI:** 10.3389/fmed.2024.1421055

**Published:** 2024-06-10

**Authors:** Zheng Zhang, Xiaojiao Tan, Haiqing Shi, Jia Zhao, Huan Zhang, Jianbo Li, Xuelian Liao

**Affiliations:** ^1^Department of Critical Care Medicine, West China Hospital, Sichuan University, Chengdu, China; ^2^Department of Critical Care Medicine, West China Tianfu Hospital of Sichuan University, Chengdu, China; ^3^Department of Critical Care Medicine, The Third People’s Hospital of Chengdu, Chengdu, China

**Keywords:** intensive care units, nosocomial infection, meta-analysis, colonization, multidrug-resistant organisms, single room

## Abstract

**Background:**

Previous studies have yielded varying conclusions regarding the impact of single-patient room design on nosocomial infection in the intensive care unit (ICU). We aimed to examine the impact of ICU single-patient room design on infection control.

**Methods:**

We conducted a comprehensive search of PubMed, Embase, the Cochrane Library, Web of Science, CNKI, WanFang Data, and CBM databases from inception to October 2023, without language restrictions. We included observational cohort and quasi-experimental studies assessing the effect of single- versus multi-patient rooms on infection control in the ICU. Outcomes measured included the nosocomial infection rate, incidence density of nosocomial infection, nosocomial colonization and infection rate, acquisition rate of multidrug-resistant organisms (MDROs), and nosocomial bacteremia rate. The choice of effect model was determined by heterogeneity.

**Results:**

Our final analysis incorporated 12 studies involving 12,719 patients. Compared with multi-patient rooms in the ICU, single-patient rooms demonstrated a significant benefit in reducing the nosocomial infection rate (odds ratio [OR]: 0.68; 95% confidence interval [CI]: 0.59, 0.79; *p* < 0.00001). Analysis based on nosocomial infection incidence density revealed a statistically significant reduction in single-patient rooms (OR: 0.64; 95% CI: 0.44, 0.92; *p* = 0.02). Single-patient rooms were associated with a marked decrease in nosocomial colonization and infection rate (OR: 0.44; 95% CI: 0.32, 0.62; *p* < 0.00001). Furthermore, patients in single-patient rooms experienced lower nosocomial bacteremia rate (OR: 0.73; 95% CI: 0.59, 0.89; *p* = 0.002) and lower acquisition rate of MDROs (OR: 0.41; 95% CI: 0.23, 0.73; *p* = 0.002) than those in multi-patient rooms.

**Conclusion:**

Implementation of single-patient rooms represents an effective strategy for reducing nosocomial infections in the ICU.

**Systematic review registration:**

https://www.crd.york.ac.uk/PROSPERO/).

## 1 Introduction

Nosocomial infections (NIs) pose a significant threat to patients admitted to intensive care unit (ICU) and are associated with the development of multiple organ dysfunction, prolonged hospitalization, and increased hospital mortality (1). A prospective cohort study involving 28 participating units across eight countries found that 18.9% of the 8,353 patients developed at least one ICU-acquired infection, leading to higher ICU mortality rates and extended lengths of stay in the ICU (2). The prevalence of multidrug-resistant organism (MDRO) infections is notably higher in the ICU than in other hospital units, due to the use of invasive devices, compromised immune statuses of patients, and increased antibiotic exposure (3). MDRO infections are particularly challenging to treat and are linked to elevated mortality rates (4). Additionally, patients colonized with MDRO face a higher risk of serious infections (5).

In the effort to prevent nosocomial colonization and infection, especially by MDROs, ICUs implement a multimodal approach to infection control, which includes antimicrobial stewardship, hand hygiene protocols, screening and isolation methods, and environmental hygiene practices (6, 7). Among these strategies, the design of hospitals and wards has emerged as a novel approach to infection control. Guidelines for ICU design recommend single-patient rooms over multi-bed rooms (8, 9). However, existing studies on the impact of ICU single-patient room design on infection control have yielded inconsistent conclusions. Some studies have shown that single-patient rooms can reduce the incidence of NIs (10–12), while others argue that the presence of single-patient rooms does not necessarily correlate with a reduction in NI rates (13–15).

To elucidate the relationship between ICU design and infection control and provide evidence for ICU ward design, we conducted a systematic review and meta-analysis of existing studies examining the impact of ICU single-patient room design on infection control.

## 2 Methods

We conducted this evidence-based analysis in accordance with the Preferred Reporting Items for Systematic Reviews and Meta-Analyses (PRISMA) 2020 statement (see Supplementary material 1 for the checklist). The study protocol was registered in the PROSPERO International prospective register of systematic reviews under the registration number CRD42023470876.

### 2.1 Search strategy

A systematic search was performed in PubMed, Embase, the Cochrane Library, Web of Science, CNKI, WanFang Data, and CBM databases from inception to October 2023. We identified potentially relevant studies primarily investigating the impact of single-patient room design on infection control in the ICU. We searched the databases using the search terms “Single,” “Private*,” “Patients’ Rooms,” “Intensive Care Units,” “Infection Control,” “Transmiss*” and “Nosocomial*.” The detailed search strategy is provided in Supplementary material 2. Two investigators independently screened titles and abstracts to identify relevant studies and subsequently evaluated full texts for inclusion. Any disagreements in the literature search were resolved through consensus.

### 2.2 Inclusion and exclusion criteria

Studies were included if they met the following criteria: (1) conducted in patients admitted to the ICU; (2) comparing single-patient rooms with multi-patient rooms in the ICU; (3) outcomes including, but not limited to, nosocomial colonization and infection rate, nosocomial infection rate, incidence density of nosocomial infection, acquisition rate of MDROs, and nosocomial bacteremia; and (4) sufficient data to calculate the odds ratio (OR).

Exclusion criteria were as follows: (1) editorial comments, case reports, conference abstracts, letters, and reviews; (2) studies defining the incorporation of single-patient rooms as part of a bundle intervention; (3) studies of single-room isolation where single-patient rooms were used as an infection control measure for already colonized or infected patients; and (4) studies not clearly stating whether the infections were acquired in the ICU.

### 2.3 Data extraction

Data extraction was independently performed by two investigators, with any discrepancies resolved through team discussion. Extracted information included first author, publication year, country of study, study design, study period, and key outcomes.

### 2.4 Quality assessment

The quality of observational cohort studies was assessed using the Newcastle-Ottawa Scale (NOS), which evaluates selection, comparability, and outcome. Studies were categorized as low (≤3 points), moderate (4–6 points), or high (≥7 points) quality. The National Heart, Lung, and Blood Institute (NHLBI) tool for before/after (pre-post) studies was used to assess the quality of nonrandomized quasi-experimental studies (16). Two investigators independently assessed the quality of included studies.

### 2.5 Statistical analysis

Data synthesis was conducted using Review Manager 5.4 version (Cochrane Collaboration, Oxford, United Kingdom). The OR for the incidence density of nosocomial infection was calculated based on the number of infections per patient-days. Mean difference and OR were used for comparing continuous and dichotomous variables, respectively. Results were presented in forest plots, with statistical significance indicated by a *p* < 0.05. Heterogeneity among studies was assessed using the inconsistency index (*I*^2^), with *I*^2^ > 50% indicating significant heterogeneity. A random-effects model was applied when significant heterogeneity was detected (*I*^2^ > 50%); otherwise, a fixed-effect model was used. One-way sensitivity analyses were conducted to assess the influence of each individual study on the combined results through removing the individual study one by one. For outcomes with significant heterogeneity, we undertook three more subgroup analysis to identify the source of heterogeneity: (i) study design; (ii) ICU type; and (iii) sample size. For outcomes with 10 or more pooled studies, we evaluated publication bias using funnel plots and Egger’s test via Stata 12.0 version (Stata Corp, College Station, TX, United States).

## 3 Results

### 3.1 Results of the search

**Figure 1:**
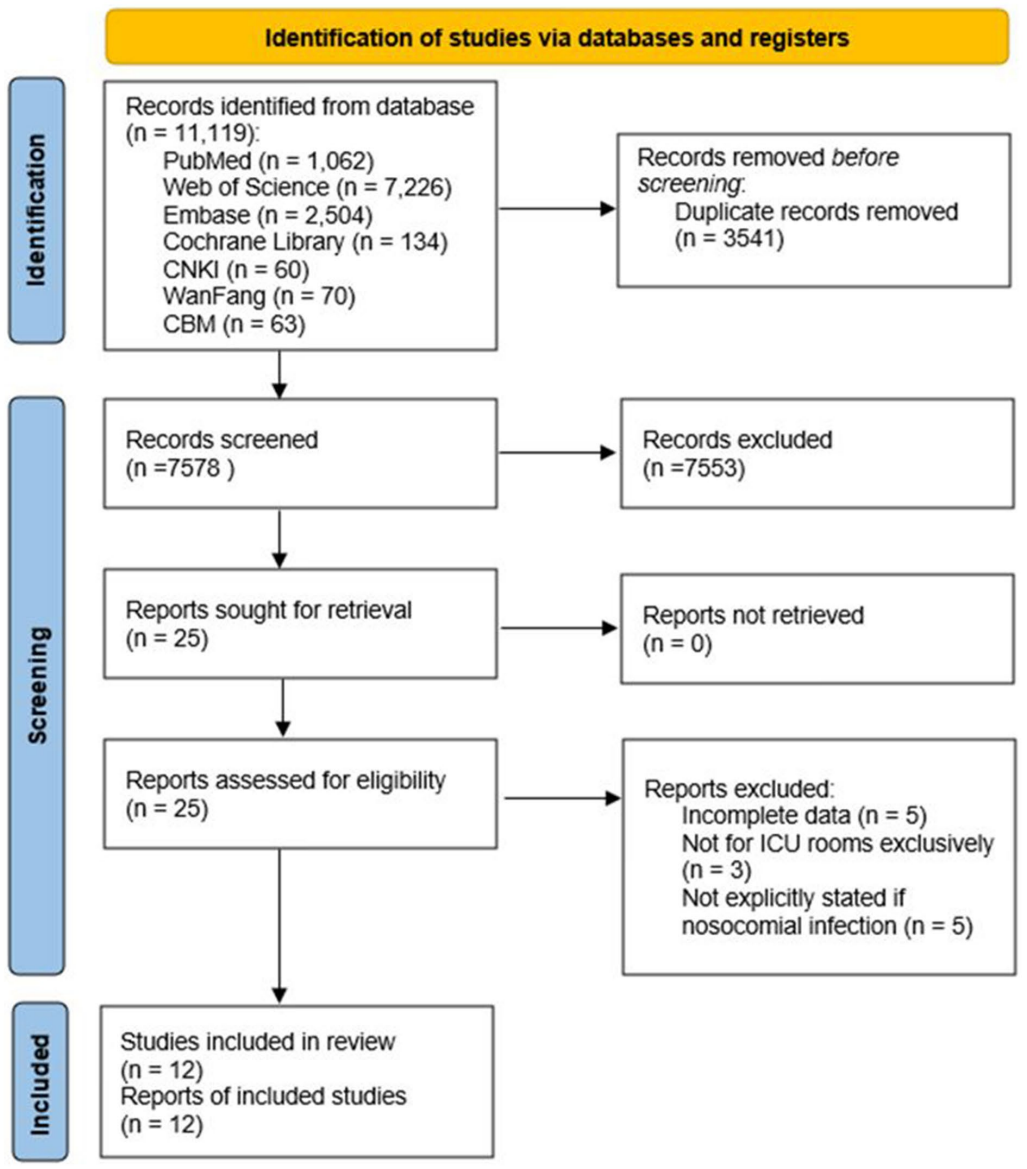
Flow diagram of study selection.

A total of 11,119 records were identified from all databases (Figure 1). After removing duplicates, the search yielded 7,578 unique publications. Titles and abstracts were screened for relevance, resulting in the assessment of 25 full-text studies. Five studies were excluded due to incomplete data, and three were excluded as they did not exclusively involve ICU rooms. Additionally, five studies were excluded as they did not explicitly specify whether the infections were nosocomial. Finally, 12 full-text articles (10, 12, 14, 17–25) were included for the pooled analysis.

### 3.2 Characteristics of included studies

Table 1 summarizes the characteristics of the included studies. The majority (9/12) (10, 12, 14, 17, 20–24) were nonrandomized quasi-experimental studies, while two (18, 19) were prospective observational studies and one (25) was retrospective observational study. All studies were conducted in ICU settings, with specific focus on neonatal ICU (3/12) (14, 20, 22) and pediatric ICU (1/12) (17). Most studies (11/12) (10, 12, 14, 17, 19–25) were conducted within a single country, with one (18) being an international multicenter study.

**Table 1:** Characteristics of the included studies.

First author, year	Country	Setting	Study design	Study period	Outcomes accessed
Ben-Abraham, 2002 (17)	Israel	Pediatric ICU	QE (pre-post intervention comparison)	1	Nosocomial infection (including bacteremia)
Bloemendaal, 2009 (18)	Netherlands, France, Spain, Portugal, Italy, and Greece	General ICU	Prospective observational study	0.25	Acquisition of MRSA
Bracco, 2007 (19)	Canada	Surgical ICU	Prospective observational study	2.5	Nosocomial bacteremia, acquisition of MRSA/*Pseudomonas aeruginosa*
Domanico, 2011 (20)	United States	Neonatal ICU	QE (pre-post intervention comparison)	2	Nosocomial sepsis
Jansen, 2021 (14)	Netherlands	Neonatal ICU	QE (pre-post intervention comparison)	4	Nosocomial infection (including bacteremia)
Jung, 2022 (10)	Republic of Korea	Medical ICU	QE (pre-post intervention comparison)	3.5	Acquisition of CRAB
Hu, 2020 (21)	China	General ICU	QE (pre-post intervention comparison)	2	MDRO infection, nosocomial infection (including bacteremia)
Lazar, 2015 (22)	Israel	Neonatal ICU	QE (pre-post intervention comparison)	8	Nosocomial bacteremia
Levin, 2011 (23)	Israel	General ICU	QE (pre-post intervention comparison)	2.5	Nosocomial bacteremia, acquisition of MDRO
Mulin, 1997 (24)	France	Surgical ICU	QE (pre-post intervention comparison)	1	Colonization or infection with A. bau-manii
Ture, 2020 (12)	Türkiye	General ICU	QE (pre-post intervention comparison)	2	Nosocomial infection, acquisition of CRAB/CAKP/CRPA
Zhang, 2018 (25)	China	General ICU	Retrospective observational study	1	MDRO infection

CRAB, carbapenem resistant *Acinetobacter baumannii*; CRKP, carbapenem resistant *Klebsiella pneumoniae*; CRPA, carbapenem resistant *Pseudomonas aeruginosa*; ICU, intensive care unit; MDRO, multidrug-resistant organisms; QE, quasi-experimental.

### 3.3 Quality assessment results

Results from the NHLBI tool (maximum total score: 12) indicated an average score of 7.89 for nonrandomized quasi-experimental studies, with the highest score being 9 and the lowest score being 5. These studies shared some common strengths, including clear research objectives, a representative inclusion of participants, well-defined intervention measures, as well as weaknesses such as not providing a description of the sample size calculation method, not detailing the blinding method, and not assessing the impact of individual-level data on the group level. All three observational studies were rated as high quality according to the NOS. Detailed quality assessment information is provided in Supplementary Tables S1, S2.

### 3.4 Nosocomial infection rate

**Figure 2:**
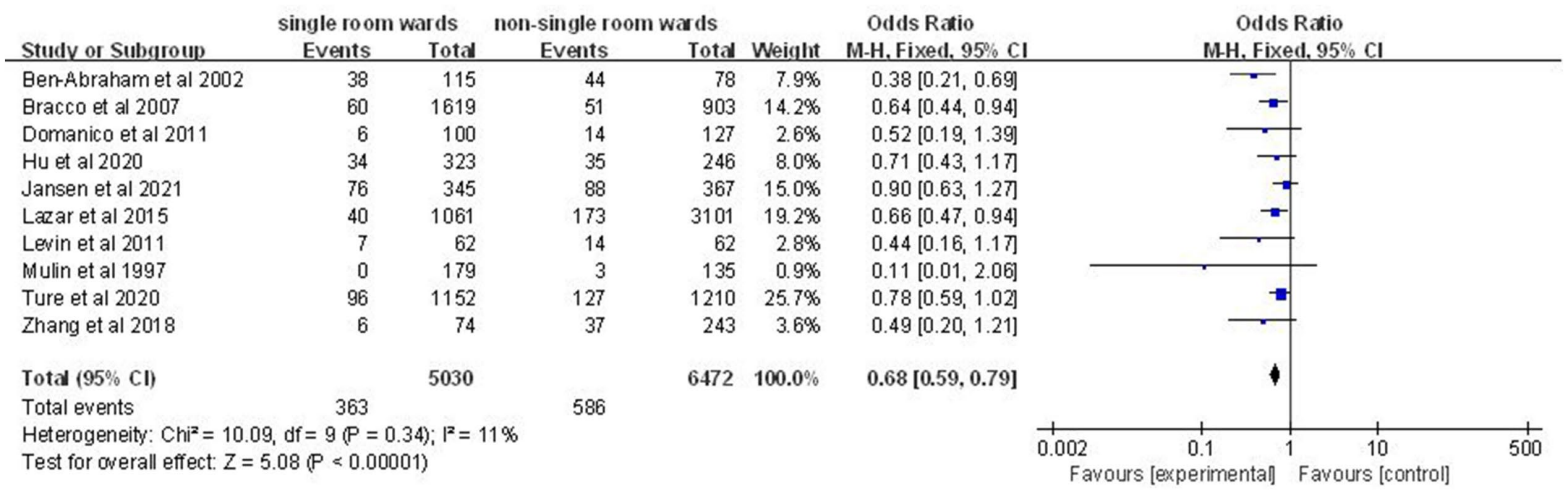
Impact of single-patient room design on nosocomial infection rate in the ICU.

Data on nosocomial infection rates were available from 10 studies involving 11,502 patients (12, 14, 17, 19–25). A significantly lower nosocomial infection rate was noted in the single-patient room group (OR: 0.68; 95% CI: 0.59, 0.79; *p* < 0.00001), with no significant heterogeneity (*I*^2^ = 11%, *p* = 0.34) (Figure 2).

### 3.5 Incidence density of nosocomial infection

**Figure 3:**
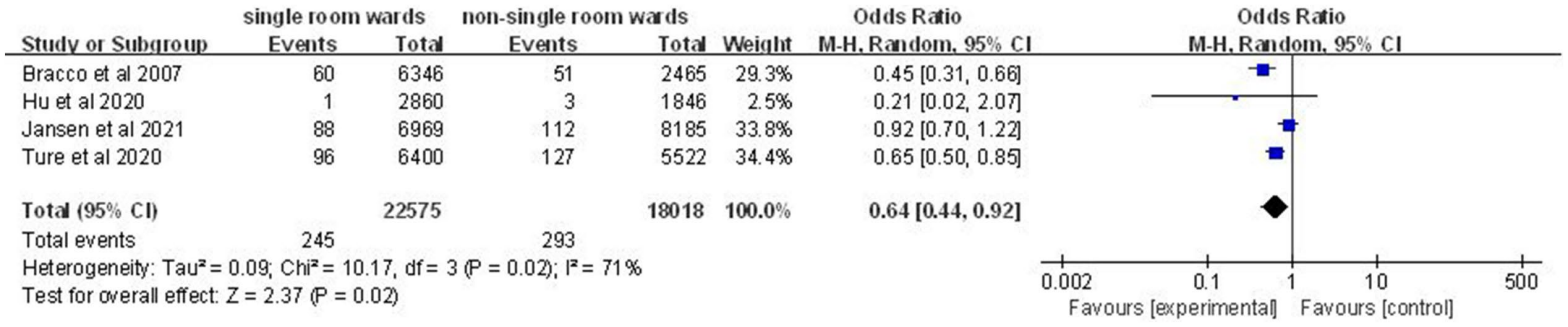
Impact of single-patient room design on incidence density of nosocomial infection in the ICU.

Four studies (12, 14, 19, 21) comprising 538 nosocomial infection episodes and 40,593 patient-days showed a significant reduction in incidence density of nosocomial infection in the single-patient room group (OR: 0.64; 95% CI: 0.44, 0.92; *p* = 0.02; Figure 3), although significant heterogeneity was observed (*I*^2^ = 71%, *p* = 0.02).

### 3.6 Nosocomial colonization and infection rate

**Figure 4:**
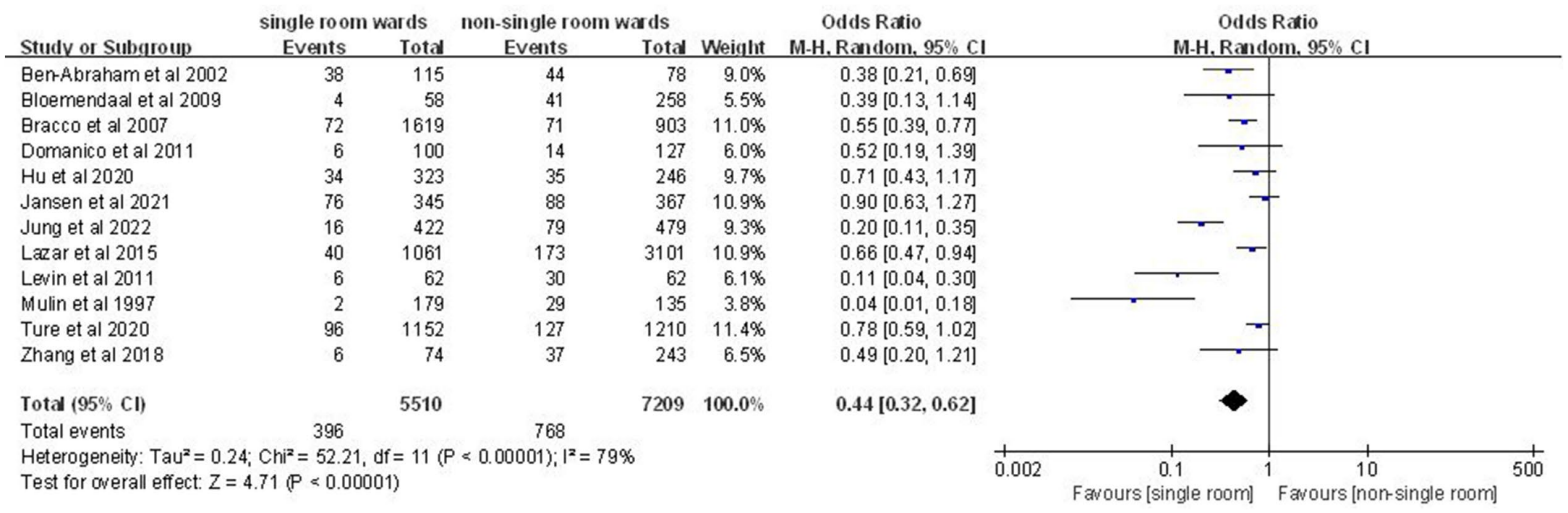
Impact of single-patient room design on nosocomial colonization and infection rate in the ICU.

Analysis of 12 studies (10, 12, 14, 17–25) involving 12,719 patients indicated a significantly lower nosocomial colonization and infection rate in the single-patient room group (OR: 0.44; 95% CI: 0.32, 0.62; *p* < 0.00001), despite significant heterogeneity (*I*^2^ = 79%, *p* < 0.00001; Figure 4).

### 3.7 Acquisition rate of MDROs

**Figure 5:**
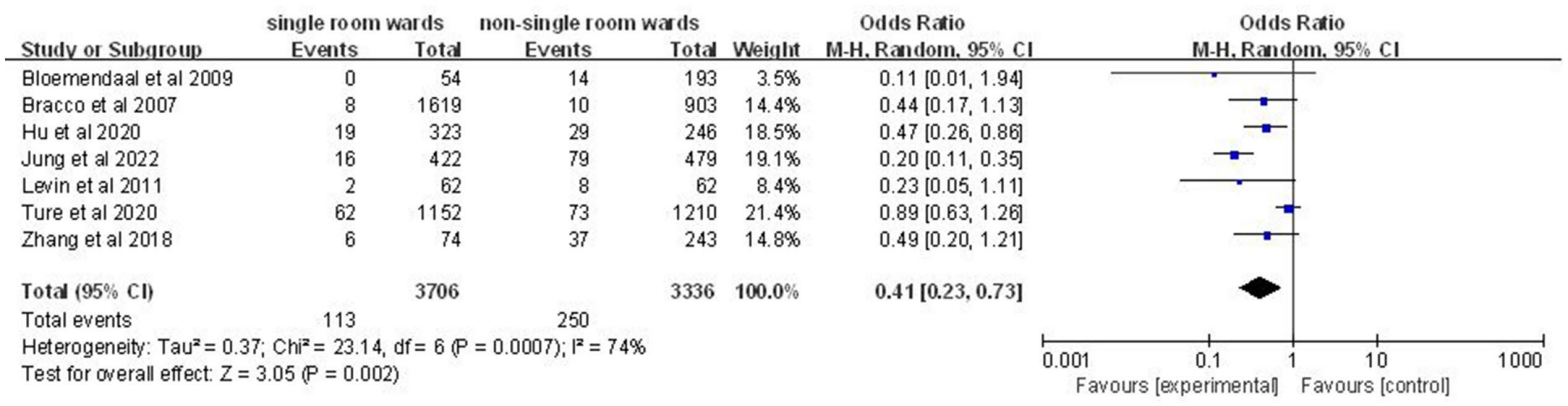
Impact of single-patient room design on acquisition rate of MDROs in the ICU.

Seven articles (10, 12, 18, 19, 21, 23, 25) involving 7,042 patients demonstrated a significantly lower acquisition rate of MDROs in the single-patient room group (OR: 0.41; 95% CI: 0.23, 0.73; *p* = 0.002; Figure 5), with significant heterogeneity (*I*^2^ = 74%, *p* = 0.0007).

### 3.8 Nosocomial bacteremia rate

**Figure 6:**
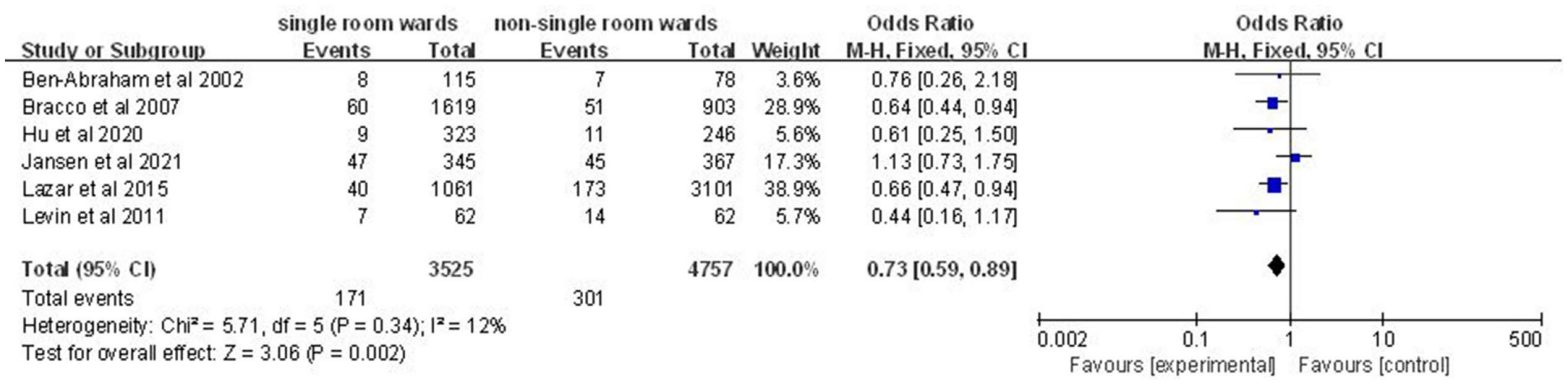
Impact of single-patient room design on nosocomial bacteremia rate in the ICU.

Analysis of six studies (14, 17, 19, 21–23) involving 8,282 patients revealed a significantly lower nosocomial bacteremia rate in the single-patient room group (OR: 0.73; 95% CI: 0.59, 0.89; *p* = 0.002; Figure 6), with no significant heterogeneity (*I*^2^ = 12%, *p* = 0.34).

### 3.9 Sensitivity analysis and subgroup analyses

One-way sensitivity analyses were conducted for various outcomes. Results indicated consistent ORs for nosocomial colonization and infection rate (Supplementary material 5, Figure S3), acquisition rate of MDROs (Supplementary material 5, Figure S4), nosocomial bacteremia rate (Supplementary material 5, Figure S5), and nosocomial infection rate (Supplementary material 5, Figure S1). However, for the incidence density of nosocomial infection, excluding data from two studies (12, 19) resulted in instability in the pooled odds ratio (Supplementary material 5, Figure S2).

The results of the subgroup analyses are detailed in Supplementary material 6. For incidence density of nosocomial infection, subgroup analysis based on study design revealed a non-significant pooled result (OR: 0.75; 95% CI: 0.53, 1.06; *p* = 0.1) with significant heterogeneity (*I*^2^ = 55%) in QE study group. When we excluded the neonatal ICU data reported by Jansen et al. in 2021 (14), the heterogeneity for the incidence density of nosocomial infection disappeared (*I*^2^ = 34%). Given the limited number of included studies, subgroup analysis based on sample size could not be applied. The results of the subgroup analysis for nosocomial colonization and infection rate were comparable to those of the pooled analysis, and substantial heterogeneity remained. The heterogeneity observed in the acquisition rate of MDROs was eliminated in the subgroup analysis based on sample size, indicating that sample size accounts for the heterogeneity.

### 3.10 Publication bias

Egger’s test and funnel plot were performed to evaluate the potential publication bias of the two outcomes. For nosocomial infection rate, funnel plot was unsymmetrical (Supplementary material 4, Figure S1) and Egger’s test also detected evidence for publication bias (*p* = 0.012, Egger’s test). For nosocomial colonization and infection rate, publication bias was observed by both the funnel plot and Egger’s test (Supplementary material 4, Figure S2; *p* = 0.009, Egger’s test).

## 4 Discussion

This study represents the first systematic review and meta-analysis assessing the impact of ICU ward design on nosocomial infection control. Our findings provide compelling evidence that adopting single-patient room design as an infection control strategy significantly reduces the incidence of nosocomial infection in ICU settings, with implications for future ICU design.

The role of ICU single-patient room design in nosocomial infection control can be elucidated through several mechanisms. First, implementing single-patient room design increases space per bed, potentially reducing overcrowding for medical staff and visitors and consequently lowering the risk of pathogen transmission through contact (26). Second, the contamination of the ward environment is a recognized cause of NIs (27). Compared with multi-bed rooms, single rooms entail less sharing of medical equipment, thereby reducing the risk of environmental contamination. Third, single-patient room design may enhance healthcare workers’ compliance with infection control practices by providing visual cues that reinforce the necessity of hand hygiene due to the spatial separation from other beds (28).

Our hypothesis focuses on the role of single-patient room design specifically in reducing NIs, not non-NIs. During literature screening, studies were excluded if they did not explicitly specify whether infections were nosocomial (11, 13, 15, 29, 30). Although most included studies defined NIs as those occurring 48 h after admission (10, 17–19, 21–25), variations existed, with one (14) extending the timeframe to 72 h and two others (12, 20) not specifying a time cutoff.

To comprehensively analyze the impact of single-patient room design on nosocomial infection control, we included patients testing positive for pathogens after 48 h of ICU admission in two outcomes, without differentiating between infection and colonization. This approach is justified as colonization often precedes infection, particularly with MDROs. Previous studies have shown that the risk of nosocomial infection is 25% with vancomycin-resistant enterococcus colonization (31), 11% with methicillin-resistant *Staphylococcus aureus* colonization (32), and up to 38% with Candida colonization (33). Our results indicate that the design of single-room wards can reduce the pathogen detection rate, including MDROs, potentially explaining the reduction in nosocomial infection rates.

Methodologically, research on the impact of single-patient room design on nosocomial infection control falls into two categories: before/after designs and observational studies. The majority of the included studies (9/12) are of a before/after design (10, 12, 14, 17, 20–24). While before/after studies involve ICU relocation and cover extended time periods, making interpretation of changes in other factors challenging, observational studies (3/12) offer better control of confounding factors as ICUs did not undergo relocation (18, 19, 25). This allowed for better control of confounding factors. However, random assignment of patients to different room types was lacking in these studies, highlighting the need for randomized controlled trials to validate the role of ICU single rooms in NIs.

While our study underscores the effectiveness of single-room ICU ward design in reducing NIs, it is essential to acknowledge the associated higher construction, operation, and maintenance costs. Hospitals must weigh these costs against potential savings from reduced hospital-acquired infections. A simulation case study by Hessam et al. suggested that cost savings from infection reduction could outweigh additional expenses in ICU settings (34). As this study is still a proof-of-concept study, it is important to consider that real-world complexities may differ, warranting cautious interpretation of the study results.

This study has several limitations. First, the analysis included both retrospective and prospective cohort designs, as well as nonrandomized quasi-experimental designs, all of which were prone to confounding bias. Second, publication bias was observed in all outcomes with 10 or more pooled studies, suggesting the presence of unpublished studies with negative results. Finally, only four studies reported incidence density data, a commonly used indicator for nosocomial infection surveillance.

## 5 Conclusion

In conclusion, our systematic review and meta-analysis demonstrate that implementing single-patient rooms in ICUs effectively reduces nosocomial infection rates, incidence density of nosocomial infection, nosocomial colonization and infection rate, acquisition rate of MDROs, and nosocomial bacteremia rate. Therefore, ICU single-patient room design is recommended for nosocomial infection control.

## Data availability statement

The original contributions presented in the study are included in the article/Supplementary material, further inquiries can be directed to the corresponding author.

## Author contributions

ZZ: Conceptualization, Data curation, Formal analysis, Methodology, Software, Writing – original draft. XT: Data curation, Formal analysis, Writing – review & editing. HS: Data curation, Formal analysis, Writing – review & editing. JZ: Formal analysis, Writing – review & editing. HZ: Formal analysis, Writing – review & editing. JL: Formal analysis, Investigation, Writing – review & editing. XL: Conceptualization, Supervision, Writing – review & editing.

## References

[1] Kollef MH Sharpless L Vlasnik J Pasque C Murphy D Fraser VJ. The impact of nosocomial infections on patient outcomes following cardiac surgery. Chest. (1997) 112:666–75. doi: 10.1378/chest.112.3.666, PMID: 9315799

[2] Alberti C Brun-Buisson C Burchardi H Martin C Goodman S Artigas A . Epidemiology of sepsis and infection in icu patients from an international multicentre cohort study. Intensive Care Med. (2002) 28:108–21. doi: 10.1007/s00134-001-1143-z, PMID: 11907653

[3] El Mekes A Zahlane K Said LA Ouafi AT Barakate M. The clinical and epidemiological risk factors of infections due to multi-drug resistant bacteria in an adult intensive care unit of university hospital center in Marrakesh-Morocco. J Infect Public Health. (2020) 13:637–43. doi: 10.1016/j.jiph.2019.08.012, PMID: 31537511

[4] Maia MO da Silveira CDG Gomes M Fernandes SES de Santana RB de Oliveira DQ . Multidrug-resistant bacteria on critically Ill patients with sepsis at hospital admission: risk factors and effects on hospital mortality. Infect. Drug Resist. (2023) 16:1693–704. doi: 10.2147/idr.S401754 36992963 PMC10042244

[5] Freedberg DE Zhou MJ Cohen ME Annavajhala MK Khan S Moscoso DI . Pathogen colonization of the gastrointestinal microbiome at intensive care unit admission and risk for subsequent death or infection. Intensive Care Med. (2018) 44:1203–11. doi: 10.1007/s00134-018-5268-8, PMID: 29936583 PMC6309661

[6] Storr J Twyman A Zingg W Damani N Kilpatrick C Reilly J . Core components for effective infection prevention and control programmes: new who evidence-based recommendations. Antimicrob Resist Infect Control. (2017) 6:6. doi: 10.1186/s13756-016-0149-9 28078082 PMC5223492

[7] Ture Z Güner R Alp E. Antimicrobial stewardship in the intensive care unit. J Intensive Med. (2023) 3:244–53. doi: 10.1016/j.jointm.2022.10.001, PMID: 37533805 PMC10391567

[8] Thompson DR Hamilton DK Cadenhead CD Swoboda SM Schwindel SM Anderson DC . Guidelines for intensive care unit design. Crit Care Med. (2012) 40:1586–600. doi: 10.1097/CCM.0b013e3182413bb2, PMID: 22511137

[9] Valentin A Ferdinande P. Recommendations on basic requirements for intensive care units: structural and organizational aspects. Intensive Care Med. (2011) 37:1575–87. doi: 10.1007/s00134-011-2300-7 21918847

[10] Jung J Choe PG Choi S Kim E Lee HY Kang CK . Reduction in the acquisition rate of carbapenem-resistant *Acinetobacter baumannii* (Crab) after room privatization in an intensive care unit. J Hosp Infect. (2022) 121:14–21. doi: 10.1016/j.jhin.2021.12.012, PMID: 34929231

[11] Liu AJ Wells A Presneill J Marshall C. Common microbial isolates in an adult intensive care unit before and after its relocation and expansion. Crit Care Resusc. (2022) 24:50–60. doi: 10.51893/2022.1.OA7, PMID: 38046844 PMC10692637

[12] Ture Z Ustuner T Santini A Aydogan S Celik İ. A comparison of nosocomial infection density in intensive care units on relocating to a new hospital. J Crit Care Med (Targu Mures). (2020) 6:175–80. doi: 10.2478/jccm-2020-0028, PMID: 32864463 PMC7430358

[13] van der Hoeven A Jansen SJ Kraakman M Bekker V Veldkamp KE Boers SA . Influence of transition from open bay units to single room units in a neonatal intensive care unit on hospital transmission of multi-drug-resistant enterobacterales. J Hosp Infect. (2023) 141:3–8. doi: 10.1016/j.jhin.2023.07.026, PMID: 37611696

[14] Jansen SJ Lopriore E Berkhout RJM van der Hoeven A Saccoccia B de Boer JM . The effect of single-room care versus open-bay care on the incidence of bacterial nosocomial infections in pre-term neonates: a retrospective cohort study. Infect Dis Ther. (2021) 10:373–86. doi: 10.1007/s40121-020-00380-9, PMID: 33355902 PMC7756131

[15] Julian S Burnham C-AD Sellenriek P Shannon WD Hamvas A Tarr PI . Impact of neonatal intensive care bed configuration on rates of late-onset bacterial sepsis and methicillin-resistant *Staphylococcus aureus* colonization. Infect Control Hosp Epidemiol. (2015) 36:1173–82. doi: 10.1017/ice.2015.144, PMID: 26108888 PMC5089903

[16] National Heart, Lung, Blood Institute. Quality Assessment Tool for Before-After (Pre-Post) Studies with No Control Group. (2014). Available at: https://www.nhlbi.nih.gov/health-topics/study-quality-assessment-tools.

[17] Ben-Abraham R Keller N Szold O Vardi A Weinberg M Barzilay Z . Do isolation rooms reduce the rate of nosocomial infections in the pediatric intensive care unit? J Crit Care. (2002) 17:176–80. doi: 10.1053/jcrc.2002.35809, PMID: 12297993

[18] Bloemendaal ALA Fluit AC Jansen WMT Vriens MR Ferry T Argaud L . Acquisition and cross-transmission of *Staphylococcus aureus* in European intensive care units. Infect Control Hosp Epidemiol. (2009) 30:117–24. doi: 10.1086/593126, PMID: 19133819

[19] Bracco D Dubois M-J Bouali R Eggimann P. Single rooms may help to prevent nosocomial bloodstream infection and cross-transmission of methicillin-resistant *Staphylococcus aureus* in intensive care units. Intensive Care Med. (2007) 33:836–40. doi: 10.1007/s00134-007-0559-5, PMID: 17347828

[20] Domanico R Davis DK Coleman F Davis BO. Documenting the nicu design dilemma: comparative patient progress in open-ward and single family room units. J Perinatol. (2011) 31:281–8. doi: 10.1038/jp.2010.120, PMID: 21072040 PMC3070087

[21] Hu X An H-Y Chen Y Liu J Li H-F Zhang Y-X . Study on Incidence of Nosocomial Infection between Single and Non-Single Wards in ICU. Chinese Journal of Nosocomiology. (2020).

[22] Lazar I Abukaf H Sofer S Peled N Leibovitz E. Impact of conversion from an open ward design paediatric intensive care unit environment to all isolated rooms environment on incidence of bloodstream infections and antibiotic resistance in Southern Israel (2000 to 2008). Anaesth Intensive Care. (2015) 43:34–41. doi: 10.1177/0310057X1504300106, PMID: 25579287

[23] Levin PD Golovanevski M Moses AE Sprung CL Benenson S. Improved ICU design reduces acquisition of antibiotic-resistant bacteria: a quasi-experimental observational study. Crit Care. (2011) 15:R211. doi: 10.1186/cc10446, PMID: 21914222 PMC3334755

[24] Mulin B Rouget C Clément C Bailly P Julliot MC Viel JF . Association of private isolation rooms with ventilator-associated *Acinetobacter baumannii* pneumonia in a surgical intensive-care unit. Infect Control Hosp Epidemiol. (1997) 18:499–503. doi: 10.2307/30141190, PMID: 9247833

[25] Zhang T Zhao Z Zhang W Zhang Y Liu B. Preliminary study of effect of single room isolation on prevention of multidrug-resistant organisms cross-infection. Chin J Nosocomiol. (2018) 28:3171–4. Available at: http://open.oriprobe.com/articles/54825750/Preliminary_study_of_effect_of_single_room_isolati.htm.

[26] Jones AR Kuschel C Jacobs S Doyle LW. Reduction in late-onset sepsis on relocating a neonatal intensive care nursery. J Paediatr Child Health. (2012) 48:891–5. doi: 10.1111/j.1440-1754.2012.02524.x, PMID: 22897216

[27] Boyce JM. Environmental contamination makes an important contribution to hospital infection. J Hosp Infect. (2007) 65:50–4. doi: 10.1016/S0195-6701(07)60015-2 17540242

[28] VanSteelandt A Conly J Ghali W Mather C. Implications of design on infection prevention and control practice in a novel hospital unit: the medical ward of the 21st century. Anthropol Med. (2015) 22:149–61. doi: 10.1080/13648470.2014.1003795 26088691

[29] van der Hoeven A Bekker V Jansen SJ Saccoccia B Berkhout RJM Lopriore E . Impact of transition from open bay to single room design neonatal intensive care unit on multidrug-resistant organism colonization rates. J Hosp Infect. (2022) 120:90–7. doi: 10.1016/j.jhin.2021.12.006, PMID: 34902498

[30] McManus AT Mason AD McManus WF Pruitt BA. A decade of reduced gram-negative infections and mortality associated with improved isolation of burned patients. Arch Surg. (1994) 129:1306–9. doi: 10.1001/archsurg.1994.01420360096013, PMID: 7986161

[31] Roghmann MC McCarter RJ Brewrink J Cross AS Morris JG. *Clostridium difficile* infection is a risk factor for bacteremia due to vancomycin-resistant enterococci (VRE) in VRE-colonized patients with acute leukemia. Clin Infect Dis. (1997) 25:1056–9. doi: 10.1086/516112, PMID: 9402356

[32] Coello R Glynn JR Gaspar C Picazo JJ Fereres J. Risk factors for developing clinical infection with methicillin-resistant *Staphylococcus aureus* (MRSA) amongst hospital patients initially only colonized with MRSA. J Hosp Infect. (1997) 37:39–46. doi: 10.1016/S0195-6701(97)90071-2, PMID: 9321727

[33] Pittet D Monod M Suter PM Frenk E Auckenthaler R. Candida colonization and subsequent infections in critically Ill surgical patients. Ann Surg. (1994) 220:751–8. doi: 10.1097/00000658-199412000-00008, PMID: 7986142 PMC1234477

[34] Sadatsafavi H Niknejad B Zadeh R Sadatsafavi M. Do cost savings from reductions in nosocomial infections justify additional costs of single-bed rooms in intensive care units? A simulation case study. J Crit Care. (2016) 31:194–200. doi: 10.1016/j.jcrc.2015.10.010, PMID: 26586445

