# Supplementary material for: Effect of single-patient room design on the incidence of nosocomial infection in the intensive care unit: a systematic review and meta-analysis
Source: Front Med (Lausanne). 2024 Jun 10;11:1421055. doi: 10.3389/fmed.2024.1421055 (PMC11194315; doi:10.3389/fmed.2024.1421055)
Supplement: Supplementary file 1 [file Data_Sheet_1.docx]

**Supplementary Material**

**Effect of single-patient room design on the incidence of nosocomial infection in the intensive care unit: A systematic review and meta-analysis**

This supplemental material has been provided by the authors to give readers additional information about their work

**Supplementary Material 1. PRISMA 2020 Checklist**

| **Section and Topic** | **Item #** | **Checklist item** | **Location where item is reported** |
| --- | --- | --- | --- |
| **TITLE** | | |  |
| Title | 1 | Identify the report as a systematic review. | 1 |
| **ABSTRACT** | | |  |
| Abstract | 2 | See the PRISMA 2020 for Abstracts checklist. | 1 |
| **INTRODUCTION** | | |  |
| Rationale | 3 | Describe the rationale for the review in the context of existing knowledge. | 2 |
| Objectives | 4 | Provide an explicit statement of the objective(s) or question(s) the review addresses. | 2 |
| **METHODS** | | |  |
| Eligibility criteria | 5 | Specify the inclusion and exclusion criteria for the review and how studies were grouped for the syntheses. | 2 |
| Information sources | 6 | Specify all databases, registers, websites, organisations, reference lists and other sources searched or consulted to identify studies. Specify the date when each source was last searched or consulted. | 2 |
| Search strategy | 7 | Present the full search strategies for all databases, registers and websites, including any filters and limits used. | 2, suppl.2 |
| Selection process | 8 | Specify the methods used to decide whether a study met the inclusion criteria of the review, including how many reviewers screened each record and each report retrieved, whether they worked independently, and if applicable, details of automation tools used in the process. | 2, Fig. 1 |
| Data collection process | 9 | Specify the methods used to collect data from reports, including how many reviewers collected data from each report, whether they worked independently, any processes for obtaining or confirming data from study investigators, and if applicable, details of automation tools used in the process. | 2 |
| Data items | 10a | List and define all outcomes for which data were sought. Specify whether all results that were compatible with each outcome domain in each study were sought (e.g. for all measures, time points, analyses), and if not, the methods used to decide which results to collect. | 2 |
|  | 10b | List and define all other variables for which data were sought (e.g. participant and intervention characteristics, funding sources). Describe any assumptions made about any missing or unclear information. | 2 |
| Study risk of bias assessment | 11 | Specify the methods used to assess risk of bias in the included studies, including details of the tool(s) used, how many reviewers assessed each study and whether they worked independently, and if applicable, details of automation tools used in the process. | 2 |
| Effect measures | 12 | Specify for each outcome the effect measure(s) (e.g. risk ratio, mean difference) used in the synthesis or presentation of results. | 2 |
| Synthesis methods | 13a | Describe the processes used to decide which studies were eligible for each synthesis (e.g. tabulating the study intervention characteristics and comparing against the planned groups for each synthesis (item #5)). | 2 |
|  | 13b | Describe any methods required to prepare the data for presentation or synthesis, such as handling of missing summary statistics, or data conversions. | 2 |
|  | 13c | Describe any methods used to tabulate or visually display results of individual studies and syntheses. | 2 |
|  | 13d | Describe any methods used to synthesize results and provide a rationale for the choice(s). If meta-analysis was performed, describe the model(s), method(s) to identify the presence and extent of statistical heterogeneity, and software package(s) used. | 2 |
|  | 13e | Describe any methods used to explore possible causes of heterogeneity among study results (e.g. subgroup analysis, meta-regression). | 2-3 |
|  | 13f | Describe any sensitivity analyses conducted to assess robustness of the synthesized results. | 2 |
| Reporting bias assessment | 14 | Describe any methods used to assess risk of bias due to missing results in a synthesis (arising from reporting biases). | 2-3 |
| Certainty assessment | 15 | Describe any methods used to assess certainty (or confidence) in the body of evidence for an outcome. | 2 |
| **RESULTS** | | |  |
| Study selection | 16a | Describe the results of the search and selection process, from the number of records identified in the search to the number of studies included in the review, ideally using a flow diagram. | 3 |
|  | 16b | Cite studies that might appear to meet the inclusion criteria, but which were excluded, and explain why they were excluded. | 3 |
| Study characteristics | 17 | Cite each included study and present its characteristics. | 3 |
| Risk of bias in studies | 18 | Present assessments of risk of bias for each included study. | 3-4, suppl.3 |
| Results of individual studies | 19 | For all outcomes, present, for each study: (a) summary statistics for each group (where appropriate) and (b) an effect estimate and its precision (e.g. confidence/credible interval), ideally using structured tables or plots. | 4, Table 1 |
| Results of syntheses | 20a | For each synthesis, briefly summarise the characteristics and risk of bias among contributing studies. | 4 |
|  | 20b | Present results of all statistical syntheses conducted. If meta-analysis was done, present for each the summary estimate and its precision (e.g. confidence/credible interval) and measures of statistical heterogeneity. If comparing groups, describe the direction of the effect. | 4-6, Figures |
|  | 20c | Present results of all investigations of possible causes of heterogeneity among study results. | 4-6 |
|  | 20d | Present results of all sensitivity analyses conducted to assess the robustness of the synthesized results. | 4-6, suppl.5 |
| Reporting biases | 21 | Present assessments of risk of bias due to missing results (arising from reporting biases) for each synthesis assessed. | 6 |
| Certainty of evidence | 22 | Present assessments of certainty (or confidence) in the body of evidence for each outcome assessed. | 4-6 |
| **DISCUSSION** | | |  |
| Discussion | 23a | Provide a general interpretation of the results in the context of other evidence. | 6 |
|  | 23b | Discuss any limitations of the evidence included in the review. | 7 |
|  | 23c | Discuss any limitations of the review processes used. | 7 |
|  | 23d | Discuss implications of the results for practice, policy, and future research. | 7 |
| **OTHER INFORMATION** | | |  |
| Registration and protocol | 24a | Provide registration information for the review, including register name and registration number, or state that the review was not registered. | 2 |
|  | 24b | Indicate where the review protocol can be accessed, or state that a protocol was not prepared. | 2 |
|  | 24c | Describe and explain any amendments to information provided at registration or in the protocol. | NA |
| Support | 25 | Describe sources of financial or non-financial support for the review, and the role of the funders or sponsors in the review. | 7 |
| Competing interests | 26 | Declare any competing interests of review authors. | 7 |
| Availability of data, code and other materials | 27 | Report which of the following are publicly available and where they can be found: template data collection forms; data extracted from included studies; data used for all analyses; analytic code; any other materials used in the review. | NA |

*From:*  Page MJ, McKenzie JE, Bossuyt PM, Boutron I, Hoffmann TC, Mulrow CD, et al. The PRISMA 2020 statement: an updated guideline for reporting systematic reviews. BMJ 2021;372:n71. doi: 10.1136/bmj.n71

For more information, visit: <http://www.prisma-statement.org/>

**Supplementary Material 2. Search strategy**

**PubMed: 1062**

1 Single[Title/Abstract]

2 Privat*[Title/Abstract]

3 Patients’ Rooms[MeSH Terms]

4 Patient's Rooms[Title/Abstract]

5 Patients Rooms[Title/Abstract]

6 Patients' Room[Title/Abstract]

7 Room, Patients'[Title/Abstract]

8 Rooms, Patients'[Title/Abstract]

9 Rooms, Patient[Title/Abstract]

10 Patient Room[Title/Abstract]

11 Patient Rooms[Title/Abstract]

12 Room, Patient[Title/Abstract]

13 1 OR 2 OR 3 OR 4 OR 5 OR 6 OR 7 OR 8 OR 9 OR 10 OR 11 OR 12

14 Intensive Care Units[MeSH Terms]

15 Intensive Care Units[Title/Abstract]

16 Intensive Care Unit[Title/Abstract]

17 Unit, Intensive Care[Title/Abstract]

18 ICU Intensive Care Units[Title/Abstract]

19 14 OR 15 OR 16 OR 17 OR 18

20 Infection Control[MeSH Terms]

21 Control, Infection[Title/Abstract]

22 transmiss*[Title/Abstract]

23 nosocomial*[Title/Abstract]

24 20 OR 21 OR 22 OR 23

25 13 AND 19 AND 24

**Embase: 2504**

#1 (Single or Privat* or Patients' Rooms or Patient's Rooms or Patients Rooms or Patients' Room or Room, Patients' or Rooms, Patients' or Rooms, Patient or Patient Room or Patient Rooms or Room, Patient).af.

#2 (Intensive Care Units or Intensive Care Unit or Unit, Intensive Care or ICU Intensive Care Units).af.

#3 (Infection Control or Control, Infection or transmiss* or nosocomial*).af.

#4 #1 AND #2 AND #3

**Web of Science 7226**

#1 (((((((((((TS=(Single)) OR TS=(Privat*)) OR TS=(Patients’ Rooms)) OR TS=(Patient's Rooms)) OR TS=(Patients Rooms)) OR TS=(Patients' Room)) OR TS=(Room, Patients')) OR TS=(Rooms, Patients')) OR TS=(Rooms, Patient)) OR TS=(Patient Room)) OR TS=(Patient Rooms)) OR TS=(Room, Patient)

#2 (((TS=(Intensive Care Units)) OR TS=(Intensive Care Unit)) OR TS=(Unit, Intensive Care)) OR TS=(ICU Intensive Care Units)

#3 (((TS=(Infection Control)) OR TS=(Control, Infection)) OR TS=(transmiss*)) OR TS=(nosocomial*)

#1 AND #2 AND #3

**The Cochrane Library: 134**

#1 MeSH descriptor: [Patients’ Rooms] explode all trees

#2 (Patient's Rooms):ti,ab,kw OR (Patients Rooms):ti,ab,kw OR (Patients' Room):ti,ab,kw OR (Room, Patients'):ti,ab,kw OR (Rooms, Patients'):ti,ab,kw

#3 (Rooms, Patient):ti,ab,kw OR (Patient Room):ti,ab,kw OR (Patient Rooms):ti,ab,kw OR (Room, Patient):ti,ab,kw

#4 #1 OR #2 OR #3

#5 MeSH descriptor: [Intensive Care Units] explode all trees

#6 (Intensive Care Units):ti,ab,kw OR (Intensive Care Units):ti,ab,kw OR (Intensive Care Unit):ti,ab,kw OR (Unit, Intensive Care):ti,ab,kw OR (ICU Intensive Care Units):ti,ab,kw

#7 #5 OR #6

#8 MeSH descriptor: [Infection Control] explode all trees

#9 (Control, Infection):ti,ab,kw OR (transmiss*):ti,ab,kw OR (nosocomial*):ti,ab,kw

#10 #8 OR #9

#11 #4 AND #7 AND #10

**CNKI (search in Chinese): 60**

The keywords presented below are phonetic transcription for Chinese characters

#1 zhongzheng(Title/Abstract/Keyword)

#2 ICU(Title/Abstract/Keyword)

#3 weizhong(Title/Abstract/Keyword)

#4 #1 OR #2 OR #3

#5 ganran(Title/Abstract/Keyword)

#6 yiyuanganran(Title/Abstract/Keyword)

#7 #5 OR #6

#8 danjian(Title/Abstract/Keyword)

#9 #4 AND #7 AND #8

**WanFang (search in Chinese): 70**

The keywords presented below are phonetic transcription for Chinese characters

#1 zhongzheng(Topic)

#2 ICU(Topic)

#3 weizhong(Topic)

#4 #1 OR #2 OR #3

#5 ganran(Topic)

#6 yiyuanganran(Topic)

#7 #5 OR #6

#8 danjian(Topic)

#9 #4 AND #7 AND #8

**CBM (search in Chinese): 63**

The keywords presented below are phonetic transcription for Chinese characters

#1 zhongzheng(Common fields: intelligence)

#2 ICU(Common fields: intelligence)

#3 weizhong(Common fields: intelligence)

#4 #1 OR #2 OR #3

#5 ganran(Common fields: intelligence)

#6 yiyuanganran(Common fields: intelligence)

#7 #5 OR #6

#8 danjian(Common fields: intelligence)

#9 #4 AND #7 AND #8

**Supplementary Material 3, Table. S1. Risk of bias assessment for before-after (Pre-Post) studies**

NHLBI assessment tool for before-after (Pre-Post) studies with no control group

| Author | Criteria 1 | Criteria 2 | Criteria 3 | Criteria 4 | Criteria 5 | Criteria 6 | Criteria 7 | Criteria 8 | Criteria 9 | Criteria 10 | Criteria 11 | Criteria 12 |
| --- | --- | --- | --- | --- | --- | --- | --- | --- | --- | --- | --- | --- |
| Ben-Abraham | 1 | 1 | 1 | 1 | CD | 1 | 1 | 0 | 1 | 1 | CD | CD |
| Domanico | 1 | 0 | 1 | CD | NA | 1 | 1 | 0 | CD | 0 | CD | CD |
| Jansen | 1 | 1 | 1 | 1 | CD | 1 | 1 | 0 | 1 | 1 | CD | CD |
| Jung | 1 | 1 | 1 | 1 | CD | 1 | 1 | 0 | 1 | 1 | CD | CD |
| Hu | 1 | 1 | 1 | 1 | CD | 1 | 1 | 0 | 1 | 1 | CD | CD |
| Lazar | 1 | 1 | 1 | 1 | CD | 1 | 1 | 0 | 1 | 1 | CD | 0 |
| Levin | 1 | 1 | 1 | 1 | CD | 1 | 1 | 0 | 1 | 1 | 1 | CD |
| Mulin | 1 | 1 | 1 | 1 | CD | 1 | 1 | 0 | 1 | 1 | 1 | CD |
| Ture | 1 | 1 | 1 | 1 | CD | 1 | 1 | 0 | 1 | 1 | CD | CD |

1, Yes; 0, No; CD, cannot determine; NA, not applicable.

1. Was the study question or objective clearly stated? 2. Were eligibility/selection criteria for the study population prespecified and clearly described? 3. Were the participants in the study representative of those who would be eligible for the test/service/intervention in the general or clinical population of interest? 4. Were all eligible participants that met the prespecified entry criteria enrolled? 5. Was the sample size sufficiently large to provide confidence in the findings? 6. Was the test/service/intervention clearly described and delivered consistently across the study population? 7. Were the outcome measures prespecified, clearly defined, valid, reliable, and assessed consistently across all study participants? 8. Were the people assessing the outcomes blinded to the participants' exposures/interventions? 9. Was the loss to follow-up after baseline 20% or less? Were those lost to follow-up accounted for in the analysis? 10. Did the statistical methods examine changes in outcome measures from before to after the intervention? Were statistical tests done that provided p values for the pre-to-post changes? 11. Were outcome measures of interest taken multiple times before the intervention and multiple times after the intervention (ie, did they use an interrupted time-series design)? 12. If the intervention was conducted at a group level (eg, a whole hospital, a community, etc.), did the statistical analysis take into account the use of individual-level data to determine effects at the group level?

**Table. S2. Quality and risk of bias assessment using the Newcastle-Ottawa Scale (NOS) for observational studies.**

| **Study ID** | **Selection** | | | | **Comparability** | **Outcome** | | | **Total**  **(9*)** |
| --- | --- | --- | --- | --- | --- | --- | --- | --- | --- |
|  | Representativeness of the exposed cohort (*) | Selection of non-exposed cohort (*) | Ascertainment of exposure (*) | Demonstration that outcome of interest was not present at start of study (*) | Comparability of cohorts (**) | Assessment of outcome (*) | Follow up long enough for outcomes to occur (*) | Adequacy of follow up (*) |  |
| Bloemendaal, 2009 | * | * | * | * |  | * | * | * | 7 |
| Bracco, 2007 | * | * | * | * | * | * | * | * | 8 |
| Zhang, 2018 | * | * | * | * | * | * | * | * | 8 |

**Supplementary Material 4, Fig. S1. Funnel plots of nosocomial infection rate**

**Fig. S2. Funnel plots nosocomial colonization and infection rate**

**Supplementary Material 5, Fig. S1. Sensitivity analysis of nosocomial infection rate**

**Fig. S2. Sensitivity analysis of incidence density of nosocomial infection**

**Fig. S3. Sensitivity analysis of nosocomial colonization and infection rate**

**Fig. S4. Sensitivity analysis of acquisition rate of MDROs**

**Fig. S5. Sensitivity analysis of nosocomial bacteremia rate**

**Supplementary Material 6, Table. S3. Subgroup analyses**

| Subgroup | Incidence density of nosocomial infection | | | | Nosocomial colonization and infection rate | | | | | Acquisition rate of MDROs | | | | |
| --- | --- | --- | --- | --- | --- | --- | --- | --- | --- | --- | --- | --- | --- | --- |
|  | Study | OR [95%CI] | P value | I² | Study | OR [95%CI] | P value | I² | Study | | OR [95%CI] | P value | I² |  |
| Total | 4 | 0.64 [ 0.44, 0.92] | 0.02 | 71% | 12 | 0.44 [ 0.32, 0.62] | ＜0.00001 | 79% | 7 | | 0.41 [ 0.23, 0.73] | 0.002 | 74% |  |
| Study design |  |  |  |  |  |  |  |  |  | |  |  |  |  |
| Cohort | 1 | 0.45 [ 0.31, 0.66] | ＜0.0001 | NA | 3 | 0.52 [ 0.39, 0.71] | ＜0.0001 | 0% | 3 | | 0.44 [ 0.23, 0.82] | 0.01 | 0% |  |
| QE | 3 | 0.75 [ 0.53, 1.06] | 0.1 | 55% | 9 | 0.42 [ 0.27, 0.64] | ＜0.0001 | 84% | 4 | | 0.40 [ 0.18, 0.92] | 0.03 | 86% |  |
| ICU type |  |  |  |  |  |  |  |  |  | |  |  |  |  |
| Pediatric or neonatal | 1 | 0.92 [ 0.70, 1.22] | 0.54 | NA | 4 | 0.64 [ 0.44, 0.91] | 0.01 | 53% | 0 | | NA | NA | NA |  |
| other types | 3 | 0.55 [ 0.40, 0.75] | 0.0002 | 34% | 8 | 0.35 [ 0.21, 0.58] | ＜0.0001 | 84% | 7 | | 0.41 [ 0.23, 0.73] | 0.002 | 74% |  |
| Sample size |  |  |  |  |  |  |  |  |  | |  |  |  |  |
| ≥1000 | 4 | 0.64 [ 0.44, 0.92] | 0.02 | 71% | 3 | 0.67 [ 0.54, 0.82] | 0.0001 | 19% | 2 | | 0.72 [ 0.39, 1.34] | 0.3 | 46% |  |
| ＜1000 | 0 | NA | NA | NA | 9 | 0.34 [ 0.20, 0.59] | 0.0001 | 82% | 5 | | 0.32 [ 0.19, 0.51] | ＜0.00001 | 32% |  |

CI = confidence interval, I² = inconsistency index, ICU = intensive care unit, MDROs = Multidrug-resistant organisms, NA = not acquired, OR = odds ratio, QE = quasi-experimental
